# Pharmacological Postoperative Pain Management for Paediatric Dental Extractions Under General Anaesthesia: A Systematic Review

**DOI:** 10.1155/prm/8569846

**Published:** 2025-01-15

**Authors:** Emily Xin Yi Ting, Sneha Sethi, Emilija Jensen, Brianna Poirier

**Affiliations:** Australian Research Centre for Population Oral Health, Adelaide Dental School, Faculty of Health and Medical Sciences, University of Adelaide, Adelaide, South Australia, Australia

**Keywords:** anaesthesia and analgesia, general anaesthesia, local anaesthesia, pain management, tooth extraction

## Abstract

General anaesthesia (GA) as a pharmacological behaviour management strategy may be indicated for dental extractions in children unable to cooperate in the dental chair. Pain is the most common postoperative complication in children following dental GA. There is conflicting evidence available on the efficacy of local anaesthetic (LA) agents for postoperative pain management following dental extraction. Therefore, this review aimed to evaluate the efficacy of different pharmacological analgesic techniques on postoperative pain following dental extractions under GA in children. A search of PubMed, Embase, Scopus and CINAHL was conducted on 17/10/2023 to identify studies eligible for inclusion in this review. Two independent reviewers performed search screening, data extraction and critical appraisal. Results were narratively described due to heterogeneity of pain assessment tools and management strategies. The search yielded 8742 results, of which 15 studies were included. Methods of pain assessment varied greatly across included studies, with 14 different pain scales used across the 15 studies. Included studies suggest preoperative oral paracetamol and oral ibuprofen as well as postoperative topical bupivacaine lowered pain scores. This review underscores the challenges in reliably assessing pain in children and highlights the necessity for age-specific validated pain assessment tools.

**Summary**

  Why this paper is important to paediatric dentists?
• Provides a comprehensive review of the effectiveness of postoperative pain management modalities for paediatric patients following dental extractions under GA.
• Raises concerns about the variation, about the range of pain management modalities as well as measurements of pain amongst paediatric populations.

## 1. Introduction

Oral diseases are highly prevalent in children and have been shown to contribute to difficulty eating, increased incidence of oral pain and school nonattendance in children [1]. Children might require dental extractions due to caries, as part of comprehensive orthodontic treatment, traumatic dental injuries, periodontal disease, prophylactic extraction prior to systemic medical treatment, over-retention of primary teeth, other oral pathology and failure of previous dental treatment [2–4]. Dental extractions are an invasive treatment, during which children may experience unpleasant and unfamiliar tastes and sounds, administration of local anaesthesia (LA) and exposure to blood [5]. Treatment under general anaesthesia (GA) may minimise stress and anxiety associated with dental extractions. However, the use of GA in very young children has been associated with physiological stressors, medical complications and although rare-potentially life-threatening adverse outcomes [6].

GA may be indicated for dental extractions in situations where children are unable to cooperate in the dental chair for psychological, mental, physical or medical reasons; have difficulty achieving adequate procedural analgesia with local anaesthetic agents; experience fear or anxiety or require emergency dental care [7]. GA as a pharmacological behaviour management strategy potentially reduces emotional stress, improves treatment efficiency and allows for more predictable dental treatment outcomes [8]. The process of GA broadly involves induction, maintenance of anaesthesia and emergence. Following the initial induction, maintenance of GA is the stage in which dental extractions are generally performed. During maintenance, blood pressure, heart rate and end-tidal carbon dioxide are monitored as markers of physiological status and in response to pain [9, 10] Fluctuation in these parameters has been observed in children undergoing extractions under GA [9].

Pain has been found to be the most common postoperative complication in children following dental treatment under GA [11–13]. Pain may be reported following dental restorations, pulpotomies, pulpectomies, extractions and stainless steel crown placements [11]. However, there is limited literature specifically related to postoperative pain following dental extractions only. Approximately 82% of adults report pain following dental extractions under LA [14], but multiple previous studies have noted the difficulty behind reliable postoperative pain assessment in the paediatric population [15–17]. Reporting of pain is dependent on cognitive ability, verbal skills and behavioural competencies [17, 18]. Examples of commonly used pain scales that are well-validated for use in children of a spread of developmental ages include The Wong and Baker Faces Pain Scale (WBFPS), validated in children aged three to 18 years old; the Visual Analogue Scale (VAS), for children aged four to 16 years old; and the Face, Legs, Activity, Cry and Consolability (FLACC) scale, validated in children aged 2 months to 7 years [19–21]. Management of postoperative pain in children plays an important role in ensuring a smooth recovery and prompt discharge, as well as a positive dental experience.

A range of strategies exist to manage postoperative pain. These include pharmacological therapies like topically applied, regional nerve block, locally infiltrated or systemically administered analgesics which may be used during the preoperative, intraoperative and/or postoperative stages. The literature is conflicted regarding the efficacy of the use of intraoperative LA [22–25]. There are also limited studies investigating the efficacy of systemic analgesics administered intraoperatively or postoperatively. Dental pain management guidelines exist for dental trauma, surgical procedures and acute dental pain for the general population [26, 27], but our search did not find guidelines specifically intended for children experiencing dental pain following extractions under GA. The existing body of research on the pharmacological management of postoperative pain following dental GA largely consists of studies older than a decade. As new drugs and protocols emerge, recent research is crucial to provide insights applicable to current clinical practices.

Site-specific infiltration of local anaesthetic agents, including field and nerve blocks, may be considered intraoperatively to decrease the physiological response to stress during dental extractions, or for postoperative pain relief. Commonly used agents include lignocaine, bupivacaine, articaine, mepivacaine and prilocaine according to the American Academy of Paediatric Dentistry. The use of LA for paediatric dental patients is discussed in the Reference Manual of Paediatric Dentistry, Chicago, III.: American Academy of Paediatric Dentistry; 2024:386-93 [28]. Each have varying properties, including duration of analgesia, but all work via sodium channel blockade and as such have limitations on safe dose. The addition of vasoconstrictors in some local anaesthetic agents, such as adrenaline, may increase the duration of analgesia and may also provide the additional benefit of reducing intraoperative bleeding if applied locally at the surgical site. Although a longer duration of analgesia may be beneficial for pain relief postoperatively, lip and cheek biting secondary to local anaesthetic spread or inadvertent field block are an unintended consequence and complication [29–31]. The evidence behind the use of infiltration or nerve-block local anaesthetic techniques during GA procedures for dental extractions is currently conflicting [16, 22–25]. Alternative methods for analgesia may be considered. This may include the administration of perioperative systemic analgesia and postoperative topical analgesia. There are various routes of administrating various systemic analgesics, such as orally, intravenously, intranasally and/or as a suppository [32]. Oral administration is commonly used as an effective and simple method of administration, while IV administration may allow for more rapid onset and more reliable dosing. Administration of analgesics rectally is usually considered when other routes of administration are unsuitable.

### 1.1. Objective

A vast array of available techniques exists aimed at reducing pain experience following dental extractions under GA. This systematic review aimed to investigate the impact of different pharmacological pain management modalities on postoperative pain following dental extractions under GA in children. It is hoped that this discussion and collation of various techniques will provide a framework for clinicians to make informed decisions when choosing analgesia methods for children who require dental extractions under GA.

## 2. Methods

### 2.1. Registration

This systematic review has been registered with the International Prospective Register of Systematic Reviews (PROSPERO), registration ID CRD42022311800. It has also been registered with the Joanna Briggs Institute (JBI) title registration. Reporting of this systematic review will be in accordance with the Preferred Reporting Items for Systematic Reviews and Meta-Analyses (PRISMA) checklist (Appendix 1) [33].

### 2.2. Systematic Literature Search

An electronic search of the literature was conducted on PubMed, Embase, Scopus and CINAHL on 17/10/2023 by one of the reviewers (BP) using a logic grid collaboratively constructed by the research team (Appendix 2). The search strategy used keywords and controlled terms, such as MeSH terms, of children, dental extraction and pain management. Studies identified were exported into Covidence systematic review software (Veritas Health Innovation, Melbourne, Australia), and duplicates were removed.

### 2.3. Study Selection

Titles and abstracts of all identified articles were screened independently by two of the researchers (ET and BP). Disagreements between the two researchers were resolved through a discussion until a consensus was reached. Full texts of articles were then retrieved and screened according to our inclusion and exclusion criteria. This screening was also performed by two independent researchers, and conflicts in opinion were discussed with a third researcher to agree upon a final decision.

### 2.4. Inclusion

Articles included were quantitative studies. Trials that involved comparison of a pharmacological analgesic technique to placebo, no treatment or another analgesic technique were eligible for inclusion. Participants included were under 18 years of age, having simple dental extractions under GA. Simple extraction refers to extractions that do not involve incision of soft tissue and/or removal of bone. We included studies that recorded postoperative pain as an outcome measure.

### 2.5. Exclusion

Study designs excluded were systematic reviews, review articles, commentaries and letters to the editor. This review excluded trials of participants requiring nonsimple, surgical extraction and extraction of a wisdom tooth or any dental procedure other than tooth extraction. Studies with no comparison group were excluded. Articles not in English were also excluded.

### 2.6. Outcome

The primary outcome for this review is postoperative pain. The timeframe for postoperative pain is defined as from when the child wakes from GA. Pain is defined as pain intensity, measured according to a pain scale, which may be self-reported, reported by a parent or guardian or reported by a health care professional.

### 2.7. Quality Assessment

Quality assessment was conducted using the Good Research for Comparative Effectiveness (GRACE) checklist (Appendix 3) [34]. The GRACE checklist was selected as the critical appraisal tool for this systematic review because it is a validated tool designed to assess observational studies on comparative effectiveness. The tool consists of 11 items, with six relating to quality of data collected and five relating to methods of the study. Two reviewers independently completed critical appraisal of each study, and any conflicts were discussed with a third reviewer so that a consensus was reached.

### 2.8. Data Extraction

A customised data extraction form was collaboratively constructed by the reviewers. The data extraction form was then completed for studies that fulfilled the selection criteria and transferred onto an excel spreadsheet through Covidence. Data collected from each study included:

1. Study characteristics: lead author's last name, year of publication, title, location the study was conducted at, study aim, study design and potential conflicts of interest
2. Population characteristics: sample size, age range of participants, gender of participants and inclusion and exclusion criteria
3. GA: method of induction and drugs used
4. Intervention: timing of postoperative pain management and use of additional analgesics
5. Measure of outcomes: intervals of recording pain scores and pain scale used
6. Study conclusion: conclusion by the authors of the study

### 2.9. Search Outcome and Evaluation

All results were narratively described due to lack of similarity across pain scales used in the studies, different drugs used to achieve analgesia, as well as timing of the analgesic intervention used (preoperatively, intraoperatively or postoperatively). A meta-analysis could not be performed due to the lack of homogenous data across and between the studies.

## 3. Results

**Figure 1:**
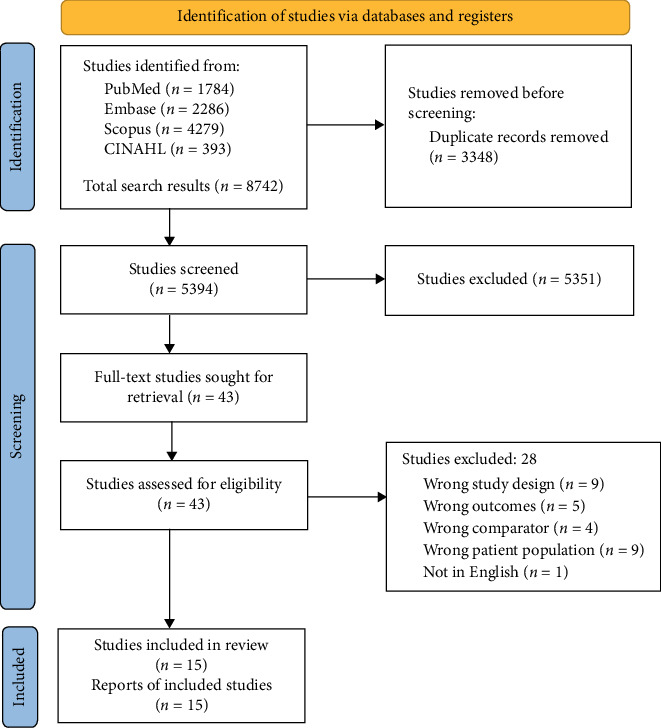
PRISMA flowchart outlining the inclusion of studies for the review.

The systematic search yielded 8742 articles, of which 3348 duplicates were removed. The remaining 5394 articles were screened independently by two researchers according to the selection criteria. Full text for 43 articles was sought for retrieval, and these then screened. A total of 15 articles satisfied the inclusion criteria and were included in this systematic review (Figure 1), while 28 were excluded (Appendix 4).

All 15 studies underwent critical appraisal by two reviewers independently using the GRACE checklist [34]. Based on the number of checklist items identified as sufficient, each study was then assigned a final score out of 11 (Appendix 5). Item three of the checklist is “Was the primary clinical outcome(s) measured objectively rather than subject to clinical judgement.” Only two articles which had participants' self-report pain using a validated pain scale scored positively for it [35, 36]. All other studies either did not use a validated pain scale or recorded pain as reported by the child's parent, a health professional or a trained researcher [16, 17, 22, 23, 25, 30, 31, 37–42]. Considering the nature of pain being inherently subjective as well as the difficulty in getting young children to understand and use pain assessment tools, scoring negatively in Item three of the checklist was not viewed as reflecting negatively against the quality of data collected in the studies. Articles generally scored better in relation to quality of data collected, but scored poorly in regards to method of the study. We also found that none of the studies restricted participants to only new initiators of treatment, specifically in relation to dental extractions.

### 3.1. Study Characteristics

Fifteen articles were included in this systematic review for analysis, 14 of which were from the United Kingdom [17, 22, 23, 25, 30, 31, 34–42] and one from the Egypt [16]. The studies were published between 1993 and 2019. This is worth noting as available anaesthetic techniques and drugs have changed substantially in the last 30 years and common practice has continually evolved. Sample sizes ranged from 24 to 210 and participants between the age range of two to 15 years. Of the 15 articles included in this review, there were 13 randomised controlled trials, one cross-sectional study and one prospective cohort study (Table 1). All included studies were parallel studies, except for the study by Anand, Wilson and Sheehy [35], which utilised a split-mouth design. This study was included because it meets our predetermined inclusion criteria, which require comparison of the intervention to a control, placebo or alternative analgesic, despite the differences in the study design. Within the included studies, GA was most commonly induced with either IV propofol or inhalation of sevoflurane and nitrous oxide, with eight studies using sevoflurane [22, 23, 35–39, 41] and seven studies using propofol [16, 22, 30, 36–39]. Other drugs used for induction of GA were halothane [16, 17, 30], methohexital [17] and isoflurane [25]. Most studies did not keep to only one method of induction for all the participants in the study.

Pain reporting approaches, pain assessment tools and timing of pain assessment varied greatly across the included studies. This is a common problem widely seen across pain-related literature and makes generalisation of results very difficult. Pain was self-reported by the child in 60% of studies [17, 22, 23, 31, 35–37, 41, 42], reported by the child's parent(s) in 33.3% of studies [22, 25, 37, 41] and either a health professional or trained observer in 66.7% of studies [16, 17, 22, 25, 30, 31, 38–41]. Of the included studies, only eight studies utilised a single approach to pain reporting, either by the child, parent(s) or a health professional [16, 23, 30, 36, 38–40]. A total of 14 different pain scales, including variations of the same pain scale, were used across the 15 studies included in this review. Five studies utilised more than one pain scale for their pain assessment [17, 25, 37, 41, 42]. One study did not utilise a pain assessment tool, but assessed pain as either present or absent [16]. Only four studies utilised a validated pain scale, either the WBFPS or the VAS for pain assessment [17, 35, 36, 42]. Assessment of pain was performed with the Toddler–Preschooler Postoperative Pain Scale in three studies [25, 40, 42]; the Hannallah Objective Pain Scale and the Central Hospital of Eastern Ontario Pain Score in two studies each [30, 38, 39, 41] and the Oucher Faces Pain Scale [41], the Distress Scale [17], the Modified Pain/Discomfort Scale [25], the Five Face Scale [22], the four-Point Scale [31], the Faces Pain Scale–revised [37] and the Morbidity Checklist and PostHospital Behaviour Questionnaire in one study each [37]. One study scored pain using an untitled pain assessment tool utilised within their local hospital context and described within their study methods [23]. This was not an externally validated tool.

### 3.2. Analgesic Techniques

A wide variety of analgesic techniques was implemented across the included studies. Out of the five studies that administered a preoperative analgesic, oral paracetamol was the most commonly used (80%). Both suppository paracetamol and diclofenac were the most commonly used intraoperative systemic analgesic across the included studies, although dosage of the drugs varied. Lignocaine was the most common LA active ingredient administered throughout the studies, but in general, a large variation in types of LA administered was noted. Including lignocaine 2% with 1:80,000 adrenaline, lignocaine 2% with 1:200,000 adrenaline, lignocaine 4% with 1:80,000 adrenaline, bupivacaine 0.25% with 1:200,000 adrenaline, bupivacaine 0.5% with 1:200,000 adrenaline and prilocaine 3% with felypressin 0.03 IU/mL (Table 2).

### 3.3. Preoperative Analgesics

The efficacy of administration of preoperative analgesics was evaluated by three clinical trials [36, 38, 41]. One study found that oral tramadol plus oral midazolam was found to reduce postoperative pain scores when compared with midazolam and placebo [41]. Another study compared postoperative pain scores of children receiving either 15 mg/kg oral paracetamol, 15 mg/kg + 5 mg/kg oral paracetamol and ibuprofen combination, 5 mg/kg oral ibuprofen alone or 20 mg/kg oral paracetamol alone [38]. This study reported no reduction of pain scores during the recovery phase across the groups when compared to the 15 mg/kg paracetamol group. However, at 15 min postoperatively, those who received ibuprofen alone or in combination with paracetamol had lower mean pain scores compared to the 15 mg/kg oral paracetamol group. A different study found administration of preoperative oral paracetamol (20 mg/kg) resulted in lower postoperative pain scores compared to no administration of analgesics [36].

### 3.4. Intraoperative Systemic Analgesics

Administration of intraoperative systemic analgesics was studied by four clinical trials [16, 30, 36, 37]. More children who received suppository paracetamol during the intraoperative phase were pain-free postoperatively compared to those who were given intraoperative topical lignocaine or not given analgesics [16]. Intraoperative rectal diclofenac was reported to reduce pain scores when compared to preoperative oral paracetamol or no analgesic administration, as assessed using the WBFPS [36]. Systemic use of fentanyl was evaluated in one study, which found that the use of IV fentanyl, alone or in combination with paracetamol, resulted in better postoperative pain management compared to participants receiving IV paracetamol alone or no systemic analgesics [37]. It should be noted that approximately 97% of participants in this study also received intraoperative infiltration of lignocaine during the procedure, although different doses of LA given were not associated with participants' self-reported pain. One study reported that IV nalbuphine and suppository diclofenac did not reduce postoperative pain scores in participants when compared with the control group receiving no analgesics [30]. The studies including rectal nonsteroidals and paracetamol are older; newer administration routes and updated, more selective nonsteroidal agents may have superseded these techniques.

### 3.5. Intraoperative Local Anaesthetics

Administration of intraoperative LA was evaluated by eight studies [16, 17, 22, 25, 35, 39, 40, 42]. There was significant heterogeneity in the technique and type of LA administration within and between these studies; techniques used include infiltration [17, 22, 25, 39], intraligamental injection [25, 35, 42] and topical application [16, 31, 40]. Three studies of different study designs investigated intraoperative infiltration of lignocaine [22, 25, 39]. Of these, one study reported no difference in postoperative pain scores when compared to placebo [22]. In the other two studies, no difference in postoperative pain scores was noted between the lignocaine groups and the no LA groups [25, 39].

One study found that prilocaine administered as an infiltration did not reduce postoperative pain scores relative to no administration of LA [17]. The VAS was used in this study, with participants ranging from age 2 to 5. However, this study reported a large proportion (47.2%) of children were unable to complete the self-reporting pain assessment, rendering these findings difficult to interpret. As a secondary outcome, this study found that children who received LA were less likely to be distressed and more likely to complete the pain assessment process, though this was not statistically assessed. Intraligamental bupivacaine delivered using a Citoject syringe provided pain relief relative to no analgesic administration, as self-assessed using the VAS [35]. One study reported intraligamental lignocaine reduced postoperative pain at 5 minute postoperatively but not at 15 min to 1 h postoperatively when compared with no LA [42]. This was assessed using the WBFPS. In a different study, children who received intraligamental lignocaine had no difference in postoperative pain scores compared to children who did not receive LA [25]. However, both the intraligamental lignocaine and control group had lower pain scores than the infiltration lignocaine group on the first night postoperatively. Topical lignocaine spray over extraction sockets was shown to effectively reduce postoperative pain when compared with no analgesic administration [16]. However, two studies found that application of bupivacaine topically over extraction sockets prior to participants emerging from GA did not improve or change pain scores on assessment when compared with the use of placebo [31, 40].

### 3.6. Postoperative Analgesics

Postoperative topical application of bupivacaine was evaluated by one study, which found rapid and effective reduction in pain scores in children who bit down on dental rolls soaked in bupivacaine compared to children who received placebo [23].

## 4. Discussion

With various techniques available for the reduction of postoperative pain following dental extractions under GA, the main objective of this review was to collate and synthesise evidence related to pharmacological pain management techniques for extractions under GA amongst paediatric populations. The findings from this systematic review suggest that preoperative administration of oral paracetamol [36] and oral ibuprofen with or without paracetamol [38] lowered postoperative pain scores in children following dental extractions under GA. An older article supported the use of intraoperative suppository paracetamol for postoperative pain management [16]. There was conflicting evidence on the efficacy of rectal diclofenac in lowering postoperative pain scores [30, 36], but these studies were not comparable between one another due to different study designs. Included articles on intraoperative LA did not support the use of infiltration of lignocaine, infiltration of prilocaine and topical bupivacaine for postoperative pain relief [17, 22, 25, 31, 39, 40]. On the other hand, intraligamental lignocaine, intraligamental bupivacaine and topical lignocaine showed reduction in postoperative pain scores [16, 35, 42]. Postoperative topical application of bupivacaine was also supported for postoperative pain relief [23].

One of the included studies found that oral tramadol plus oral midazolam reduced postoperative pain scores compared to midazolam and placebo [41]. However, the FDA released an announcement in April 2017, restricting the use of tramadol in children under 12 years old [43]. Following this, the Society for Paediatric Anaesthesia in New Zealand and Australia (SPANZA) also provided recommendations cautioning its use in children [44]. As a result, oral tramadol is no longer a recommended analgesic technique for the purpose of paediatric dental treatment.

This review highlights the immense variation in pain measurement and management amongst paediatric dentistry practices. The number of different analgesic techniques used across the studies reflects the lack of standardised pain relief guidelines for simple/nonsurgical dental extractions under GA. Within studies performed in the United Kingdom alone, 14 different methods of pain measurement were used. Multiple studies discussed the difficulty of pain assessment in young children, especially following GA. The heterogeneity of pain assessment across the studies meant that pain experience was incomparable between participants of different studies, and age differences across populations made the application of pain scoring systems internally inconsistent as well. This emphasises the importance of utilising a validated pain scale for assessment with paediatric populations. While the WBFPS and VAS are examples of pain assessment tools validated for use in children, they are valid amongst different age ranges; the WBFPS is best suited for younger children than the VAS, compromising accuracy of comparisons across these measures [19]. Additionally, it is important to consider the limitations of using general pain scales to assess site-specific pain, pain following dental extractions is often localised and a generalised pain scale designed to assess the overall pain may not accurately reflect pain intensity in a site-specific context. Another factor to consider is how the timing of pain assessment may affect reliability of self-reported pain scores. Participants still recovering from the effects of GA may be disoriented, which could compromise their ability to accurately report their pain. These considerations contribute to the difficulty in undertaking reproducible research that may be reliably generalised across different developmental groups in children.

### 4.1. Limitations

Due to the heterogeneous nature of the data including type of intervention, study design and outcome measure across included studies, a meta-analysis of the data extracted was deemed inappropriate. Contributing to an overall lack of studies investigating the same type of intervention, limited evidence was available on the efficacy of each intervention. There is a need for further high-quality randomised controlled trials with comparable interventions and pain assessment to establish the efficacy of various analgesic techniques on postoperative pain management. The administration of addition analgesics was not controlled for in multiple studies, which also made interpretation of postoperative pain scores difficult. Despite the comprehensive search across four databases, there was a limited geographic spread of papers identified. Fourteen of the 15 studies included were carried out in the United Kingdom, and one study was from the Egypt. While the similarity of patient population may make the studies more comparable, interpretation of data collected from this review may not apply to the global paediatric population.

In conclusion, this systematic review demonstrates the wide variety of analgesic techniques used for postoperative pain management following paediatric dental extractions under GA. There was insufficient evidence on the efficacy of each intervention for a guideline to be developed from this review. The variation in study design, pain assessment protocol and analgesic technique made comparison between included studies difficult. This systematic review highlights the difficulty in performing reliable pain assessment in the paediatric population and emphasises the importance of age-specific validated pain assessment tools. The use of validated pain scales in future studies could increase comparability of pain assessment results between different studies. Administration of various analgesics should be controlled for to reliably assess the efficacy of the invention administered. There is a need for more robust randomised controlled trials of similar study designs so data can be compared across more geographic locations..

## Figures and Tables

**Table 1:** Study characteristics of the included studies.

Study	Country	Study aim	Study design	Participant characteristics	Main findings
Alohali et al. [37]	United Kingdom	To compare the use of fentanyl alone, paracetamol alone and paracetamol with fentanyl	Prospective	*N* = 143 (4–10 years, 55.9% male and 44.1% female)	Approximately 75% of children reported postoperative pain following primary teeth extractions under GA^||^, use of IV paracetamol and fentanyl reduced immediate postoperative self-reported pain
Anand, Wilson and Sheehy [35]	United Kingdom	To assess the effectiveness of ITR^†^ LA^§^ compared with no LA^§^	RCT⁣^∗^	*N* = 30 (10–13 years, 43.3% male and 56.7% female)	ITR^†^ bupivacaine was useful and safe for postoperative pain management among children having permanent teeth extractions under GA^||^
Andrzejowski and Lamb [31]	United Kingdom	To assess the efficacy of bupivacaine-soaked dental rolls compared with placebo	RCT⁣^∗^	*N* = 120 (5–12 years, gender not specified)	Children exposed to the bupivacaine and saline swabs reported the same postoperative pain measurements; familiarising children with pain scales prior to GA^||^ improved ability to self-report pain
Coulthard et al. [22]	United Kingdom	To investigate the effect of IFL^‡^ lignocaine compared to placebo	RCT⁣^∗^	*N* = 139 (4–12 years, 52.5% male and 47.5% female)	Intraoperative LA^§^ was not found to be effective for reducing postoperative pain or distress in children after oral surgery under GA^||^
Elhakim [16]	Egypt	To evaluate the efficacy of postoperative topical lignocaine and rectal paracetamol compared with no analgesia	RCT⁣^∗^	*N* = 60 (4–11 years, gender not specified)	Children who received topical lignocaine had significantly lower pain scores with no need for postoperative analgesia than those who received no analgesia
Gazal and Mackie [38]	United Kingdom	To compare pain relief with paracetamol alone, ibuprofen alone and paracetamol and ibuprofen in combination	RCT⁣^∗^	*N* = 201 (2–10 years, gender not specified)	There were significant decreases in mean pain and distress scores for both ibuprofen alone and paracetamol/ibuprofen combination groups compared to the control group
Greengrass, Andrzejowski and Ruiz [23]	United Kingdom	To assess the efficacy of topical bupivacaine compared to placebo	RCT⁣^∗^	*N* = 24 (7–15 years, 41.7% male and 58.3% female)	Bupivacaine with adrenaline soaked swaps significantly reduced pain in children 10 min postoperative
Leong, Roberts and Ashley [25]	United Kingdom	To investigate the efficacy ITR^†^ and IFL^‡^ LA^§^ compared to no LA^§^	RCT⁣^∗^	*N* = 54 (2–6 years, gender not specified)	ITR^†^ LA^§^ was beneficial and well tolerated in managing postoperative pain
Littlejohn et al. [30]	United Kingdom	To compare the analgesic properties of nalbuphine and diclofenac with a control	RCT⁣^∗^	*N* = 60 (≥ 3 years, 50% male and 50% female)	Both IV nalbuphine or diclofenac suppositories showed low median pain scores, thus, may be clinically significant
McWilliams and Rutherford [39]	United Kingdom	To assess the efficacy of IFL^‡^ LA^§^ compared to no LA^§^	RCT⁣^∗^	*N* = 85 (< 6 years, gender not specified)	No difference in pain scores noted between the IFL^‡^ LA^§^ and no LA^§^ group, however, adjunct use of LA^§^ can be considered with GA^||^, as it helps reduce bleeding
Noble et al. [17]	United Kingdom	To assess efficacy of IFL^‡^ LA^§^ compared to no LA^§^	RCT⁣^∗^	*N* = 100 (3–14 years, 55% male and 45% female)	Pain assessment was unsatisfactory, as a large proportion of children were unable to complete it. Less early postoperative distress was observed after prilocaine infiltration
O'Donnell et al. [36]	United Kingdom	To compare effect of preoperative paracetamol, intraoperative diclofenac and no analgesia	Cross-sectional study	*N* = 210 (3–12 years, 51% male and 49% female)	Diclofenac is a better preemptive analgesic as compared to paracetamol for dental extractions under GA^||^
Quirke, Bhaskar and Choonara [40]	United Kingdom	To study the efficacy of intraoperative topical bupivacaine compared to placebo	RCT⁣^∗^	*N* = 48 (4–13 years, gender not specified)	Intraoperative topical bupivacaine with adrenaline showed no significant reduction in postoperative pain
Roelofse and Payne [41]	United Kingdom	To evaluate effect of oral tramadol plus midazolam compared with midazolam alone	RCT⁣^∗^	*N* = 60 (4–7 years, 53.3% male and 46.7% female)	Tramadol plus midazolam was effective in alleviating pain and no deleterious effects were observed, thus, a reliable and effective option
Sammons et al. [42]	United Kingdom	To evaluate the use of ITR^†^ LA^§^ compared to no LA^§^	RCT⁣^∗^	*N* = 85 (2–5 years, 55.3% male and 44.7% female)	ITR^†^ lignocaine only provided short term pain relief at 5 min postoperatively

⁣^∗^RCT = randomised control trial.

^†^ITR = intraligamental injection.

^‡^IFL = infiltration.

^§^LA = local anaesthetic.

^||^GA = general anaesthesia.

**Table 2:** Pain management strategies.

	Preoperative analgesics	Intraoperative systemic analgesics		Postoperative analgesic	Main finding
		Systemic	Local		
Alohali et al. [37]	—	Control: no systemic analgesic Experimental 1: IV^†^ paracetamol Experimental 2: IV^†^ fentanyl Experimental 3: IV^†^ paracetamol + fentanyl	IFL^§^ lignocaine 2% with adrenaline 1:80,000 Each pt received either: 0.0 mL, 0.55 mL, 0.73 mL, 1.1 mL, 1.47 mL, 1.65 mL, 2.2 mL	All pt received either: No analgesic oral paracetamol Oral ibuprofen oral paracetamol and ibuprofen	Systemic use of fentanyl and paracetamol reduced postoperative pain

Anand, Wilson and Sheehy [35]	—	All pts received either one of: IV^†^ ketorolac IV^†^ alfentanyl Suppository diclofenac sodium suppository alone Suppository diclofenac sodium with IV^†^ alfentanyl Suppository paracetamol	Control side: no LA⁣^∗^ Experimental side: ITR^‡^ using a Citoject syringe′ bupivacaine, 0.5% with 1:200,000 epinephrine 0.2 mL per root	10 children: oral paracetamol 5 children: oral ibuprofen	63% of children reported better pain control on the ITR^‡^ bupivacaine side

Andrzejowski and Lamb [31]	—	—	Swabs soaked in 5 mL of solution placed topically Control: saline Experimental 1: bupivacaine, 0.25% with epinephrine 1:200,000	All groups: suppository diclofenac 1 mg/kg and for any further discomfort, paracetamol	No difference in pain scores between using topical bupivacaine and saline

Coulthard et al. [22]	All groups: 15 mg/kg paracetamol elixir and EMLA^”^ topical paste on the dorsum of both hands 1 h preoperatively		Control: IFL^§^ placebo^∼^ Experimental 1: IFL^§^ 2 mL lignocaine, 2% with 1:200,000 epinephrine	All groups: paracetamol elixir to take home with appropriate dosing information	No difference in mean pain scores between the LA⁣^∗^ and placebo groups

Elhakim [16]	—	Experimental 2: suppository paracetamol 10 mg/kg	Control: no analgesic Experimental 1: 4 mg/kg topical lignocaine^@^	—	The topical lignocaine group reported less pain compared with the paracetamol and control groups

Gazal and Mackie [38]	All groups: topical EMLA⁣^∗^ cream applied to both hands 1 h before induction Control: oral paracetamol (15 mg/kg) Experimental 1: oral paracetamol/ibuprofen combination (15 mg/kg + 5 mg/kg) Experimental 2: oral ibuprofen (5 mg/kg) Experimental 3: oral paracetamol (20 mg/kg)	—	—	—	Use of ibuprofen and ibuprofen/paracetamol combination resulted in lower pain scores than compared to normal- or high-dose paracetamol

Greengrass, Andrzejowski and Ruiz [23]	—	—	—	Dental rolls soaked in 7 mL solution to bite down on Control: placebo^∼^ Experimental 1: bupivacaine 0.25% with 1:200,000 adrenaline	Postoperative topical bupivacaine achieved relief of postoperative discomfort

Leong, Roberts and Ashley [25]	—	All groups: suppositories diclofenac sodium 1–2 mg/kg and paracetamol 20–40 mg/kg	Lignocaine, 2% with epinephrine 1: 80,000 Control: no LA⁣^∗^ Experimental 1: IFL^§^ 0.5 mL each quadrant requiring extraction with self-aspirating syringe^$^ Experimental 2: ITR^‡^ 0.2 mL mesiobuccal PDL^||^ of each tooth to be extracted using an intraligamental syringe^$^	—	The ITR^‡^ LA⁣^∗^ and no LA⁣^∗^ group had no difference in pain scores compared to each other, but lower pain scores on the first night compared to the IFL^§^ LA⁣^∗^ group. Pain scores were the same across all groups at all other time points of observation.

Littlejohn et al. [30]	—	Control: no analgesic Experimental 1: IV^†^ nalbuphine hydrochloride 0.3 mg/kg Experimental 2: suppository diclofenac 12.5 mg to a dose of −2 mg/kg		Patients complaining of pain or felt to be in pain by the recovery staff: offered paracetamol	No differences between the analgesic effects of either IV^†^ nalbuphine or diclofenac suppositories compared with control

McWilliams and Rutherford [39]	Both groups: oral paracetamol (20 mg/kg) and ibuprofen (5 mg/kg)	Children who refused oral premedication: rectal diclofenac suppositories (1 mg/kg) under anaesthesia	Control: no LA⁣^∗^ Experimental: IFL^§^ lignocaine 4% with 1:80,000 adrenaline	—	No difference in postoperative pain

Noble et al. [17]	—		Control: no LA⁣^∗^ Experimental: IFL^§^ prilocaine 3% and felypressin (0.03 IU/mL) 15–45 mg for each tooth^^^	—	Pain assessment was unsatisfactory; only 13% of distressed children and 68% of nondistressed children were able to complete the pain assessment

O'Donnell et al. [36]	Control: no pain relief Experimental 1: oral paracetamol, 20 mg/kg	Experimental 2: rectal diclofenac, 25 mg (children under 12 kg-half the dose)	—	—	Diclofenac reduced pain more than paracetamol or no analgesia Paracetamol reduced pain more than no analgesia

Quirke, Bhaskar and Choonara [40]	—	—	Gamgee swabs soaked with 5 mL of solutions and placed over sockets Control: Placebo^∼^ Experimental: bupivacaine, 0.25% with 1:200,000 adrenaline (5 mcg/mL)	Both groups: offered oral paracetamol suspension	Bupivacaine LA⁣^∗^ showed no reduction of postoperative pain compared to placebo

Roelofse and Payne [41]	Both groups: oral midazolam (0.5 mg/kg) (maximum dose 7.5 mg) Control: placebo (saline drops) Experimental: tramadol drops (1.5 mg/kg)	—	—	Both groups: paracetamol syrup 120 mg (5 mL), as needed	Tramadol plus midazolam resulted in less pain compared to midazolam plus placebo

Sammons et al. [42]	Both groups: Single preoperative doses of ibuprofen (10 mg/kg) and paracetamol (20 mg/kg)		Lignocaine 2% with adrenaline 1:80,000; 0.15 mL per tooth, maximum of 2 mL Control: no LA⁣^∗^ Experimental: ITR^‡^	Both groups: codeine as required (500 μg–1 mg·kg^−1^ per dose 4–6 hourly)	ITR^‡^ lignocaine reduced pain at 5 min postoperatively, but not at 15 min to 1 h postoperative

⁣^∗^LA = local anaesthetic.

^†^IV = intravenous.

^‡^ITR = intraligamental injection.

^§^IFL = infiltration.

^||^PDL = periodontal ligament.

^”^EMLA = eutectic mixture of local anaesthetics, 2.5% lignocaine and 2.5% prilocaine.

^^^Total dose administered depended on size of the child and number of teeth infiltrated.

^$^Self-aspirating syringe (AspijectÂ self-aspirating dental syringe, Denmark) with 30G 21 mm needles (CarÂ¬puleÂ, Heraeus Kulzer).

′Intraligamentary syringes (CitojectÂ, Heraeus Kulzer) with 30G (X-Short) 10 mm needle (HypoÂ, Dentsply, USA).

^@^Using a metered spray, each spray delivering 10 mg of solution.

^∼^0.9% Sodium chloride solution.

## Data Availability

No new data were generated by this research, and all data supporting the findings of this study are available within the paper and its Supporting Information.

## Supporting Information

Supporting Information 1 Appendix 1: PRISMA 2020 checklist.

Supporting Information 2 Appendix 2: Logic grid.

Supporting Information 3 Appendix 3: GRACE checklist.

Supporting Information 4 Appendix 4: Excluded studies.

Supporting Information 5 Appendix 5: Critical Appraisal Results.
