# Supplementary material for: Pharmacological Postoperative Pain Management for Paediatric Dental Extractions Under General Anaesthesia: A Systematic Review
Source: Pain Res Manag. 2025 Jan 15;2025:8569846. doi: 10.1155/prm/8569846 (PMC11753856; doi:10.1155/prm/8569846)
Supplement: Supporting Information 2 — Appendix 2: Logic grid. [file 8569846.f2.docx]

Logic Grid

**PubMed**

| Children | Dental extraction | Pain management |
| --- | --- | --- |
| Child[mh] OR child*[tiab] OR juvenile[tiab] OR Adolescent[mh] OR adolescen*[tiab] OR teen*[tiab] OR young adult*[tiab] OR youth*[tiab] OR young people[tiab] OR young person[tiab] OR high school[tiab] OR primary school[tiab] | Tooth extraction[mh:noexp] OR dental extraction*[tiab] OR exodontia*[tiab] OR premolar*[tiab] OR molar*[tiab] OR tooth[tiab] OR teeth[tiab] OR extraction*[tiab] OR dental[tiab] | Analgesics[mh] OR opioid*[tiab] OR Opiate[tiab] OR Local anaesthetic*[tiab] OR Local anaesthesia[tiab] OR Analgesia[tiab] OR Ice pack[tiab] OR Analgesic*[tiab] OR Fentanyl[tiab] OR Alfentanil[tiab] OR Oxycodone[tiab] OR Tramadol[tiab] OR morphine[tiab] OR paracetamol[tiab] OR ibuprofen[tiab] OR acetaminophen[tiab] OR NSAID*[tiab] OR non-steroidal anti-inflammator*[tiab] OR Cox-2 inhibitor*[tiab] OR cyclooxygenase-2 inhibitor*[tiab] OR Non-medical therapy[tiab] OR conservative treatment[tiab] OR Pain management[tiab] OR pain relief[tiab] OR general anaesthe*[tiab] |

**Embase**

| Children | Dental extraction | Pain management |
| --- | --- | --- |
| Child.sh OR adolescent.sh OR child*.ti,ab OR minor*.ti,ab OR juvenile.ti,ab OR adolescen*.ti,ab OR teen*.ti,ab OR young adult*.ti,ab OR youth*.ti,ab OR young people.ti,ab OR young person.ti,ab OR high school.ti,ab OR primary school.ti,ab | Tooth extraction.sh OR dental extraction*.ti,ab OR exodont*.ti,ab OR premolar*.ti,ab OR molar*.ti,ab OR extraction*.ti,ab OR teeth.ti,ab OR tooth.ti,ab OR dental.ti,ab | Analgesic agent.sh OR analgesia.sh OR opiate.sh OR postoperative analgesia.sh oR opioid*.ti,ab OR Opiate*.ti,ab OR Local anaesthe*.ti,ab OR anaesthe*.ti,ab OR Analgesi*.ti,ab OR Ice pack.ti,ab OR Fentanyl.ti,ab OR Alfentanil.ti,ab OR Oxycodone.ti,ab OR Tramadol.ti,ab OR morphine.ti,ab OR paracetamol.ti,ab OR ibuprofen.ti,ab OR acetaminophen.ti,ab OR NSAID*.ti,ab OR non-steroidal anti-inflammator*.ti,ab OR Cox-2 inhibitor*.ti,ab OR cyclooxygenase-2 inhibitor*.ti,ab OR Non-medical therapy.ti,ab OR conservative treatment.ti,ab OR pain management.ti,ab OR pain relief.ti,ab OR general anaesthesia.ti,ab |

**Scopus**

| Children | Dental extraction | Pain management |
| --- | --- | --- |
| Child* OR minor OR juvenile OR adolescen* OR teen* OR "young adult*" OR youth OR "young people" OR "young person" OR "primary school" | Exodont* OR Premolar* OR Molar* OR Extraction* OR "tooth extraction*" OR "dental extraction*" OR "tooth removal" OR tooth OR teeth OR dental | Opioid* OR opiate* OR "Local anaesthe*" OR "Ice pack" OR Analgesi* OR Fentanyl OR Alfentanil OR Oxycodone OR Tramadol OR morphine OR paracetamol OR ibuprofen OR acetaminophen OR NSAID* OR "non-steroidal anti-inflammator*" OR "Cox-2 inhibitor*" OR "cyclooxygenase-2 inhibitor*" OR "Non-medical therapy" OR "conservative treatment" OR "Pain management" OR "pain relief" OR "general anaesthesia" |

**CINAHL**

| Children | Dental extraction | Pain management |
| --- | --- | --- |
| MH child OR TI (Child* OR Minor* OR Juvenile* OR Adolescent* OR adolescen* OR teen* OR "young adult*" OR youth* OR "young people" OR "young person*" OR "high school*" OR "primary school*") OR  AB (Child* OR Minor* OR Juvenile* OR Adolescent* OR adolescen* OR teen* OR "young adult*" OR youth* OR "young people" OR "young person*" OR "high school*" OR "primary school*") | MH "tooth extraction" OR TI (Exodonti* OR premolar* OR molars* OR extraction* OR tooth OR teeth OR "tooth extraction*" OR "teeth extraction*" OR "dental extraction*" OR "tooth removal" OR dental) OR AB (Exodonti* OR premolar* OR molars* OR extraction* OR tooth OR teeth OR "tooth extraction*" OR "teeth extraction*" OR "dental extraction*" OR "tooth removal" OR dental) | MH analgesics OR TI (Opioid* OR Opiate* OR "Local anaesthe* OR Analgesi* OR anaesthe* OR "Ice pack" OR Analgesi* OR Fentanyl OR Alfentanil OR Oxycodone OR Tramadol OR morphine OR paracetamol OR ibuprofen OR acetaminophen OR NSAID* OR "non-steroidal anti-inflammator*" OR "Cox-2 inhibitor*" OR "cyclooxygenase-2 inhibitor*" OR "Non-medical therapy" OR "conservative treatment" OR "Pain management" OR "general anaesthe*") OR AB (Opioid* OR Opiate* OR "Local anaesthe* OR Analgesi* OR anaesthe* OR "Ice pack" OR Analgesi* OR Fentanyl OR Alfentanil OR Oxycodone OR Tramadol OR morphine OR paracetamol OR ibuprofen OR acetaminophen OR NSAID* OR "non-steroidal anti-inflammator*" OR "Cox-2 inhibitor*" OR "cyclooxygenase-2 inhibitor*" OR "Non-medical therapy" OR "conservative treatment" OR "Pain management" OR "general anaesthe*") |
