# Supplementary material for: Pharmacological Postoperative Pain Management for Paediatric Dental Extractions Under General Anaesthesia: A Systematic Review
Source: Pain Res Manag. 2025 Jan 15;2025:8569846. doi: 10.1155/prm/8569846 (PMC11753856; doi:10.1155/prm/8569846)
Supplement: Supporting Information 4 — Appendix 4: Excluded studies. [file 8569846.f4.docx]

| Title | Authors | Exclusion reason |
| --- | --- | --- |
| Ketamine is effective in decreasing the incidence of emergence agitation in children undergoing dental repair under sevoflurane general anesthesia | Abu-Shahwan, I.; Chowdary, K. | Wrong outcomes |
| The incidence of post-extraction pain and analgesic usage in children | Acs, G.; Moore, P. A.; Needleman, H. L.; Shusterman, S. | Wrong study design |
| Anaesthesia, pain and recovery profiles in children following dental extractions | Akl, N.; Sommerfield, A.; Slevin, L.; Drake-Brockman, T. F.; Wong, S.; Winters, J. C.; Ungern-Sternberg, B. S. V.; Sommerfield, D. | Wrong comparator |
| Management of pain and anxiety in the paediatric patient | Alberts, I. L. | Wrong outcomes |
| Effect of pre-operative administration of ibuprofen in children undergoing extraction for reducing post-operative pain: a double-blind randomised controlled clinical trial | Alshami, A. A.; Alhamed, S. A.; Almahri, J. R.; AlSaggabi, F. A.; Alshahrani, A. S. | Wrong patient population |
| Comparison of pre-emptive ibuprofen, paracetamol, and placebo administration in reducing post-operative pain in primary tooth extraction | Baygin, O.; Tuzuner, T.; Isik, B.; Kusgoz, A.; Tanriver, M. | Wrong patient population |
| A high proportion of children undergoing dental treatment under general anesthesia experience postoperative dental pain | Brignardello-Petersen, R. | Wrong study design |
| Postoperative discomfort of dental rehabilitation under general anesthesia | Cantekin, K.; Yildirim, M. D.; Delikan, E.; Çetin, S. | Wrong outcomes |
| Descriptive study of perioperative analgesic medications associated with general anesthesia for dental rehabilitation of children | Carter, L.; Wilson, S.; Tumer, E. G. | Wrong patient population |
| Lidocaine use for pain management during paediatric dental rehabilitation under general anaesthesia | El Batawi, H. Y. | Wrong outcomes |
| Pain reported by children after dental extractions under general anaesthesia: a pilot study | Fung, D. E.; Cooper, D. J.; Barnard, K. M.; Smith, P. B. | Wrong comparator |
| Does preoperative paracetamol reduce pain after dental treatment? A randomized controlled double-blind study | Fux-Noy, A.; Bendahan, Y.; Ungar, E.; Shmueli, A.; Halperson, E.; Ram, D.; Moskovitz, M. | Wrong patient population |
| Efficacy of Preemptive Analgesia on Postoperative Pain Control in Children Who Underwent Full-Mouth Dental Rehabilitation Under General Anesthesia: A Randomized Controlled Clinical Trial | Keles, S.; Kocaturk, O.; Demir, P. | Wrong outcomes |
| Reiki therapy for postoperative oral pain in pediatric patients: pilot data from a double-blind, randomized clinical trial | Kundu, A.; Lin, Y.; Oron, A. P.; Doorenbos, A. Z. | Wrong outcomes |
| Effect of Intrapapillary Local Anesthetic on Postoperative Pain Following Dental Treatment Under General Anesthesia in Pediatric Patients | Lipp, K.; Casamassimo, P.; Griffen, A.; Smiley, M.; Bryant, J.; Draper, J.; Kumar, A. | Wrong patient population |
| Post-operative pain | Lyons, H. J. | Wrong study design |
| [Fentiazac for postoperative pain and inflammation in children. Double blind clinical trial against placebo] | Mano Azul, A.; Mourão, F. | Not in English |
| Factors associated with children's perception of pain following dental treatment | Mathias, F. B.; Cademartori, M. G.; Goettems, M. L. | Wrong comparator |
| Postextraction pain relief in children: A clinical trial of liquid analgesics | Moore, P. A.; Acs, G.; Hargreaves, J. A. | Wrong patient population |
| Audit of analgesia following dental extractions in children | Muthukrishnan, S.; Jonker, G.; Fletcher, V.; Hivey, S. | Wrong study design |
| Fiorinal with codeine as a postoperative analgesic in dental surgery | Nelson, R. | Wrong patient population |
| Pain relief for paediatric dental chair anaesthesia: current practice in a community dental clinic | S. Jürgens; R. S. Warwick; P. J. Inglehearn; D. S. Gooneratne | Wrong patient population |
| Intraoperative local anaesthesia for reduction of postoperative pain following general anaesthesia for dental treatment in children and adolescents | Parekh, S.; Gardener, C.; Ashley, P. F.; Walsh, T. | Wrong study design |
| Ibuprofen for Pain Control in Children: New Value for an Old Molecule | Poddighe, D.; Brambilla, I.; Licari, A.; Marseglia, G. L. | Wrong study design |
| Comparison of preoperative ibuprofen, acetaminophen, and placebo administration on the parental report of postextraction pain in children | Primosch, R. E.; Nichols, D. L.; Courts, F. J. | Wrong patient population |
| Use of local anesthesia during dental rehabilitation with general anesthesia: A survey of dentist anesthesiologists | Townsend, J. A.; Hagan, J. L.; Smiley, M. | Wrong study design |
| A holistic approach for postoperative pain management in children receiving dental treatment under general anesthesia: A randomized clinical trial | Liu B.; Wang P.; Zhang Y.; Feng C.; Wang J.; Zhang H. | Not in English |
| Postoperative Pain of Pediatric Patients Undergoing Dental Treatment under General Anesthesia Visiting a General Hospital: A Cross-Sectional Study | Kakti A.; Abumelha R.K.; Alajmi A.M.; Dagriri L.K.; Alkodari L.A.; Fares M.J.; Cicciu M.; Minervini G. | Wrong intervention |
