# Supplementary material for: Pharmacological Postoperative Pain Management for Paediatric Dental Extractions Under General Anaesthesia: A Systematic Review
Source: Pain Res Manag. 2025 Jan 15;2025:8569846. doi: 10.1155/prm/8569846 (PMC11753856; doi:10.1155/prm/8569846)
Supplement: Supporting Information 5 — Appendix 5: Critical Appraisal Results. [file 8569846.f5.docx]

### Critical Appraisal Results

| **Citation** | **Q1** | **Q2** | **Q3** | **Q4** | **Q5** | **Q6** | **Q7** | **Q8** | **Q9** | **Q10** | **Q11** | **Score** |
| --- | --- | --- | --- | --- | --- | --- | --- | --- | --- | --- | --- | --- |
| Sammons 2007 | Y | Y | N | Y | Y | Y | N | Y | Y | Y | Y | 9 |
| Roelofse 1999 | Y | Y | N | N | Y | Y | N | Y | Y | Y | N | 7 |
| Quirke 2005 | Y | Y | N | Y | Y | N | N | Y | N | Y | N | 6 |
| O'Donnell 2007 | Y | Y | Y | Y | Y | N | N | Y | N | Y | N | 7 |
| Noble 1994 | N | N | N | N | Y | Y | N | Y | N | Y | N | 4 |
| McWilliams 2007 | N | Y | N | Y | Y | N | N | Y | Y | Y | N | 6 |
| Littlejohn 1996 | Y | Y | N | Y | Y | Y | N | Y | Y | Y | N | 8 |
| Leong 2007 | Y | Y | N | Y | Y | N | N | Y | Y | Y | Y | 8 |
| Greengrass 1998 | Y | N | N | N | Y | Y | N | N | N | Y | N | 4 |
| Gazal 2007 | Y | Y | N | N | Y | Y | N | N | Y | Y | N | 6 |
| Elhakim 1993 | Y | N | N | N | Y | Y | N | N | N | Y | N | 4 |
| Coulthard 2006 | Y | Y | N | N | Y | N | N | Y | N | Y | N | 5 |
| Andrzejowski 2002 | Y | Y | N | N | Y | N | N | Y | Y | Y | N | 6 |
| Anand 2005 | Y | Y | Y | Y | Y | Y | N | Y | N | Y | N | 8 |
| Alohali 2019 | N | N | N | N | N | N | N | Y | N | Y | N | 2 |
